# Mendelian randomization reveals the causal links between miRNAs and rheumatoid arthritis

**DOI:** 10.1097/MD.0000000000045527

**Published:** 2025-10-31

**Authors:** Zehong Wei, DongXu Chen, LianFa Li, Junping Yang, Ying Wang

**Affiliations:** a Department of Nephrology, YuDu Hospital of Traditional Chinese Medicine, YuDu, Jiangxi, China; b Department of Pathology, Affiliated Hospital of Jiangxi University of Chinese Medicine, Nanchang, Jiangxi, China; c School of Clinical Medicine, Jiangxi University of Chinese Medicine, Nanchang, Jiangxi, China.

**Keywords:** biomarker, causal effect, circulating microRNAs, Mendelian randomization, rheumatoid arthritis

## Abstract

Rheumatoid arthritis (RA) is a chronic autoimmune disease characterized by persistent joint inflammation and progressive structural damage, with early diagnosis remaining a significant clinical challenge. Circulating microRNAs (miRNAs) have emerged as promising biomarkers for disease diagnosis, prognosis, and therapeutic response due to their critical roles in gene regulation. However, the specific miRNAs causally involved in RA pathogenesis remain largely unidentified. We conducted a 2-sample Mendelian randomization (MR) analysis using summary-level data from the largest available genome-wide association study of circulating cis-miRNA expression quantitative trait loci (cis-miR-eQTLs) and RA genome-wide association study summary statistics. The inverse variance weighted method served as the primary analytical approach, supplemented by comprehensive sensitivity analyses including Cochran Q test, MR-Egger intercept test, MR-PRESSO, and leave-one-out analysis to ensure result robustness. Additionally, we performed target gene prediction, gene ontology and kyoto encyclopedia of genes and genomes enrichment analyses, and druggable analysis to explore the underlying biological mechanisms and therapeutic potential of the causal miRNAs. Our MR analysis identified 8 circulating miRNAs with significant causal associations with RA risk. Notably, hsa-miR-130a-3p (*P* = 6.5332 × 10^−5^, OR = 1.0720, 95% CI = 1.0360–1.1092) emerged as a key risk factor, while hsa-miR-204-5p (*P* = 6.2123 × 10^−4^, OR = 0.9707, 95% CI = 0.9543–0.9874) demonstrated a protective effect. Bioinformatics analyses revealed that hsa-miR-130a-3p may modulate the TGF-β, Hippo, and mTOR signaling pathways by interacting with competing endogenous RNAs (ceRNAs) such as H19 and regulating hub proteins including TNF, UBB, PPARG, and TGFBR1. Resveratrol and flufenamic acid were identified as candidate therapeutic agents targeting its downstream pathways. Conversely, hsa-miR-204-5p was predicted to influence the AMPK, cGMP-PKG, and cAMP signaling pathways via ceRNAs like NEAT1 and NORAD, affecting key proteins such as BCL2, SIRT1, and HMGA2, with cilostazol, melatonin, and curcumin identified as potential modulators. This study provides novel causal evidence implicating hsa-miR-130a-3p and hsa-miR-204-5p in RA pathogenesis. These findings highlight their potential as circulating biomarkers for early diagnosis and risk assessment, as well as therapeutic targets for miRNA-based intervention strategies, thereby offering valuable insights for advancing precision medicine in RA management.

## 1. Introduction

Rheumatoid arthritis (RA) is a chronic, systemic autoimmune disease characterized by persistent synovial inflammation, progressive joint destruction, and diverse systemic manifestations, all contributing to significantly reduced quality of life and increased risks of disability and mortality in affected patients.^[1]^ Early diagnosis and prompt initiation of treatment are critical to halting disease progression, minimizing joint damage, and improving long-term prognosis.^[2]^ However, early-stage RA remains challenging to diagnose due to its clinical heterogeneity and overlapping symptoms with other arthritic disorders.^[3]^ Currently, the 2010 American College of Rheumatology/European League Against Rheumatism (ACR/EULAR) classification criteria serve as the primary diagnostic criteria for RA, incorporating serological markers such as rheumatoid factor and anti-cyclic citrullinated peptide antibody.^[4]^ Despite their widespread clinical use, the sensitivity and specificity of these biomarkers remain suboptimal, often leading to delayed or missed diagnoses and suboptimal disease control.^[3]^ Consequently, there is an urgent need to identify novel and more reliable biomarkers for the early diagnosis and therapeutic evaluation of RA.

MicroRNAs (miRNAs), a class of small noncoding RNAs approximately consisting of 19 to 25 nucleotides, have emerged as critical post-transcriptional regulators of gene expression involved in immune regulation, inflammation, and autoimmunity.^[5,6]^ Due to their remarkable stability in various biofluids such as serum, plasma, and synovial fluid, miRNAs have attracted considerable interest as promising diagnostic, prognostic, and therapeutic biomarkers for RA.^[7]^ Previous studies employing microarray and RNA-seq technologies have revealed differential miRNA expression profiles in peripheral blood mononuclear cells and synovial tissues of RA patients, providing novel insights into disease pathogenesis.^[8–10]^ Nevertheless, most of these studies are observational in nature and cannot fully exclude the influence of confounding factors such as age, sex, smoking, and body mass index, nor can they establish definitive causal relationships between miRNA dysregulation and RA susceptibility or progression.

Mendelian randomization (MR) is an analytical method that leverages genetic variants as instrumental variables (IVs) to estimate the causal effect of an exposure on an outcome,^[11]^ thereby complementing the limitations of conventional observational studies. In MR studies, genetic variants are randomly allocated at conception and are generally independent of environmental and behavioral confounders, thereby substantially reducing confounding bias and the risk of reverse causation.^[12–14]^ Therefore, MR analysis can be regarded as a natural tool approximating randomized controlled trials, providing a powerful approach for causal inference.^[15]^ In recent years, MR has been widely applied to explore the causal effects of various biomarkers, metabolites, and circulating molecules on complex diseases, including autoimmune disorders.

In this study, we conducted a 2-sample MR analysis by integrating data from large-scale genome-wide association studies (GWAS) and the largest cis-miRNA expression quantitative trait loci (cis-miR-eQTL) datasets. To our knowledge, this is the first large-scale 2-sample MR study to systematically assess the causal role of circulating miRNAs in RA using summary-level data from these resources. Furthermore, we performed comprehensive bioinformatics analyses, including target gene prediction and functional enrichment, to elucidate the potential biological mechanisms linking RA-associated miRNAs to disease pathogenesis. By identifying miRNAs with putative causal roles in RA development, our findings may contribute to the discovery of novel biomarkers and therapeutic targets, thereby advancing the field of precision medicine in RA management.

## 2. Materials and methods

### 2.1. Study design

**Figure 1.**
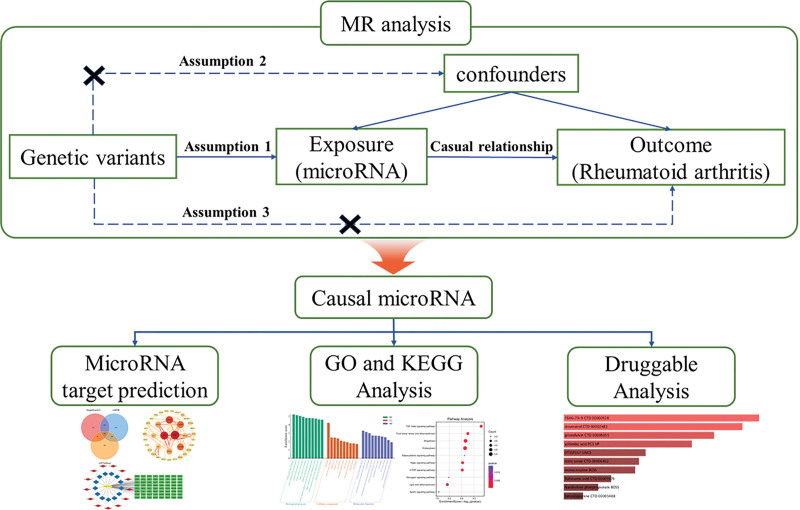
The flowchart of this study. Cis-miR-eQTL data were used as exposures, and RA GWAS data as outcomes. Genetic variants served as instrumental variables to assess the causal effects of circulating microRNAs on RA. Cis-miR-eQTL = cis-miRNA expression quantitative trait loci, GWAS = genome-wide association study, RA = rheumatoid arthritis.

The overall design and analytical workflow of this study are illustrated in Figure 1. To investigate the potential causal relationship between circulating miRNAs and RA, we extracted IVs from the largest study of cis-miR-eQTLs to perform a 2-sample MR.^[16]^ In MR analysis, genetic variants used as IVs must fulfill 3 key assumptions to ensure valid results^[17]^: the relevance assumption: genetic variants must be strongly correlated with the exposure; the independent assumption: genetic variants should not be associated with confounding factors; the exclusion restriction assumption: genetic variants influence the outcome exclusively through the exposure. Following the primary MR analysis, we conducted a series of downstream bioinformatics analyses, including the prediction and construction of the protein–protein interaction (PPI) network and competing endogenous RNA (ceRNA) regulatory network, as well as gene ontology (GO) and kyoto encyclopedia of genes and genomes (KEGG) enrichment analyses and druggable analysis. These complementary analyses aimed to explore the potential biological mechanisms through which the identified miRNAs may influence RA susceptibility and progression, thereby providing theoretical support for future experimental validation.

### 2.2. Data acquisition

We obtained the miRNA eQTL data from the largest publicly available cis-miR-eQTLs study to date, which investigated miRNA expression profiling of whole blood-derived RNA from 5239 individuals in the Framingham Heart Study and identified 5269 cis-miR-eQTLs associated with 76 mature miRNAs.^[16]^ These genetic variants associated with miRNA expression were utilized as instruments to investigate the causal impact of miRNAs on RA. RA genome-wide association studies (GWAS) data were obtained from FinnGen R12 (https://www.finngen.fi/fi [accessed on May 25, 2025]) as outcome data, which included 16,314 RA patients and 315,115 healthy individuals, and identified 21,323,666 independent SNPs (single nucleotide polymorphisms). The large sample sizes of both the exposure dataset (FHS, n = 5239) and the outcome dataset (FinnGen, cases = 16,314; controls = 315,115) provided sufficient statistical power for reliable causal inference. All data were from European-ancestry individuals. In addition, all data used in this study were sourced from publicly available databases and did not include any personal or identifiable information. No new human data were gathered, and ethical approval was not necessary.

### 2.3. Selection criteria of IVs

To maintain a balance between statistical rigor and the availability of sufficient IVs, we adopted the default threshold (*P* < 1 × 10^−5^), as implemented in the commonly used TwoSampleMR package, to perform MR analysis.^[18]^ Additionally, we conducted linkage disequilibrium (LD) clumping based on the European-ancestry reference data from the 1000 Genomes Project to ensure independence among IVs. The LD clumping parameters were set with a clumping window of 10,000 kb and an r² threshold of <0.1 to minimize potential bias caused by strong LD.^[18]^ Subsequently, to eliminate the potential influence of weak IVs, *F*-statistics were calculated for each SNP to assess their statistical strength, and only strong IVs (*F*-statistics >10) were retained.^[19]^ We utilized the LDtrait function from the LDlinkR package (https://github.com/CBIIT/LDlinkR) to retrieve trait or disease associations of SNPs from the GWAS Catalog (accessed on May 31, 2025), and excluded SNPs related to confounding factors affecting RA, such as age, sex, obesity, smoking and other potential confounders.

### 2.4. Mendelian randomization analysis

The MR analyses were performed using R (version 4.5.0) and the TwoSampleMR package (https://github.com/MRCIEU/TwoSampleMR [accessed on May 26, 2025]), with inverse variance weighted (IVW) as the primary analytical method.^[20,21]^ To further validate the reliability of the results, we conducted multiple sensitivity analyses to detect potential heterogeneity and pleiotropy.^[22]^ Cochran *Q* test and MR-Egger regression were used to assess heterogeneity and pleiotropy, respectively, with *P*-values >.05 indicating no significant evidence of either.^[23]^ Additionally, the MR-pleiotropy residual sum and outlier (MR-PRESSO) method was applied to detect and correct for pleiotropy by identifying and removing outlier SNPs.^[22]^ Furthermore, a leave-one-out analysis was performed to assess whether the MR analysis was driven or biased by a single SNP.

### 2.5. MiRNA target prediction and regulatory network visualization

We predicted miRNA target genes using TargetScanHuman 8.0^[24]^ (https://www.targetscan.org/vert_80/ [accessed on June 2, 2025]), miRDB^[25]^ (https://mirdb.org/ [accessed on June 2, 2025]), and miRTarBase^[26]^ (https://awi.cuhk.edu.cn/~miRTarBase/miRTarBase_2025/php/index.php [accessed on June 2, 2025]), identified upstream lncRNAs and circRNAs via miRNet^[27]^ (https://www.mirnet.ca/ [accessed on June 2, 2025]) and ENCORI^[28]^ (https://rnasysu.com/encori/ [accessed on June 2, 2025]), and extracted overlapping targets across databases using R (version 4.5.0) to identify reliable potential targets. The overlapping target genes of miRNAs were imported into the STRING^[29]^ database (https://string-db.org [accessed on June 3, 2025]) with the study species limited to “Homo sapiens,” the minimum interaction score set at 0.400, and all other parameters kept at their default values to construct the PPI network. Subsequently, the PPI analysis data and lncRNA-miRNA-mRNA network data were imported into Cytoscape^[30]^ v.3.10.1 to visualize the PPI network and the lncRNA-miRNA-mRNA regulatory network. Notably, the node sizes and colors in the PPI network were adjusted according to degree values to highlight hub genes.

### 2.6. GO and KEGG analysis

We used R (version 4.5.0) to convert the overlapping target genes of miRNAs into their corresponding Ensembl gene IDs (https://www.ensembl.org/index.html [accessed on June 4, 2025]). The R packages “clusterProfiler”^[31]^ and “org.Hs.e.g..db.” were used to conduct GO and KEGG pathway enrichment analyses. A *P*-value <.05 was set as the significance threshold to identify the most significant GO terms and pathways. The “enrichplot,” “ggplot2” R packages, and an online platform^[32]^ were used to visualize enrichment results.

### 2.7. Druggable analysis

To further investigate druggable genes related to RA and identify promising candidate drugs, we first uploaded the overlapping target genes of miRNAs to the DGIDB^[33]^ database (https://dgidb.org/ [accessed on June 7, 2025]) to screen for valuable druggable genes. Subsequently, we analyzed the druggable genes using the DSigDB tool on the Enrichr^[34]^ online platform (https://maayanlab.cloud/Enrichr/ [accessed on June 7, 2025]) to screen for potential small-molecule drugs or targeted therapies that may modulate RA-related pathogenic pathways.

## 3. Result

### 3.1. MR Analysis reveals the causal relationships between circulating miRNAs and RA

**Figure 2.**
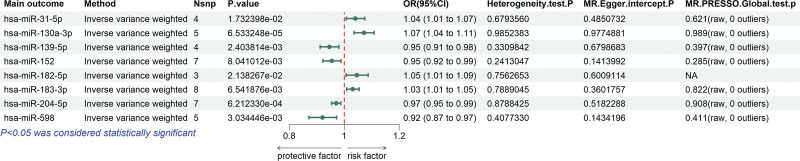
The forest plot of causal relationships between miRNAs and RA using the IVW method. Green horizontal lines depict the 95% CI for each miRNA. Green dots represent the point estimates of ORs, with dots positioned to the right of the red vertical line indicating risk factors, and those to the left indicating protective factors. 95% CI = 95% confidence intervals, IVW = inverse variance weighted, miRNA = microRNAs, ORs = odds ratios, RA = rheumatoid arthritis.

**Figure 3.**
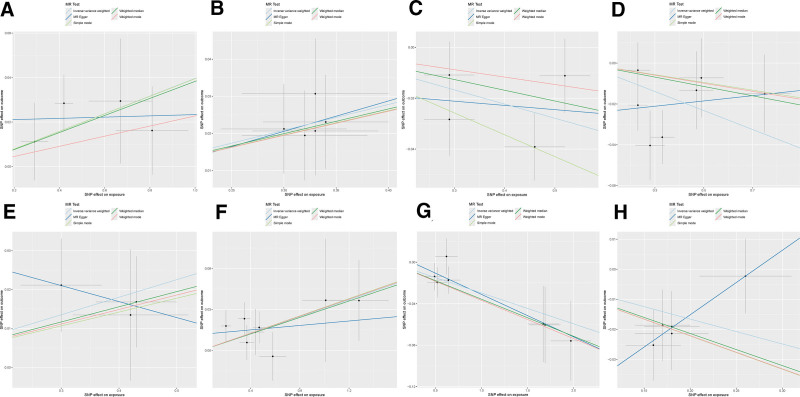
The scatter plot of analysis assessing the causal relationship between miRNAs and RA. (A) hsa-miR-31-5p, (B) hsa-miR-130a-3p, (C) hsa-miR-139-5p, (D) hsa-miR-152, (E) hsa-miR-182-5p, (F) hsa-miR-183-3p, (G) hsa-miR-204-5p, (H) hsa-miR-598. MR = Mendelian randomization, RA = rheumatoid arthritis, miRNA = microRNAs.

Based on the selection criteria for IVs, a total of 46 SNPs associated with 8 miRNAs were identified as strong IVs (*F*-statistics >10). Detailed information on these IVs is provided in Table S1, Supplemental Digital Content, https://links.lww.com/MD/Q532. Further MR analysis of the causal relationships between these circulating miRNAs and RA identified 4 risk factors (IVW: OR >1, *P* <.05) and 4 protective factors (IVW: OR < 1, *P* <.05). The results are presented in the forest plot (Fig. 2) and the scatter plot (Fig. 3).

The IVW method identified 4 circulating miRNAs exhibiting causal associations with an increased risk of RA: hsa-miR-31-5p (*P* = 1.7324 × 10^−2^, OR = 1.0404, 95% CI = 1.0070–1.0750), hsa-miR-130a-3p (*P* = 6.5332 × 10^−5^, OR = 1.0720, 95% CI = 1.0360–1.1092), hsa-miR-182-5p (*P* = 2.1383 × 10^−2^, OR = 1.0461, 95% CI = 1.0067–1.0871), hsa-miR-183-3p (*P* = 6.5419 × 10^−3^, OR = 1.0314, 95% CI = 1.0087–1.0547). In addition, 4 miRNAs exhibited protective causal effects against RA: hsa-miR-139-5p (*P* = 2.4038 × 10^−3^, OR = 0.9458, 95% CI = 0.9124–0.9805), hsa-miR-152 (*P* = 8.0410 × 10^−3^, OR = 0.9546, 95% CI = 0.9224–0.9880), hsa-miR-204-5p (*P* = 6.2123 × 10^−4^, OR = 0.9707, 95% CI = 0.9543–0.9874), hsa-miR-598 (*P* = 3.0344 × 10^−3^, OR = 0.9205, 95% CI = 0.8714–0.9723).

**Figure 4.**
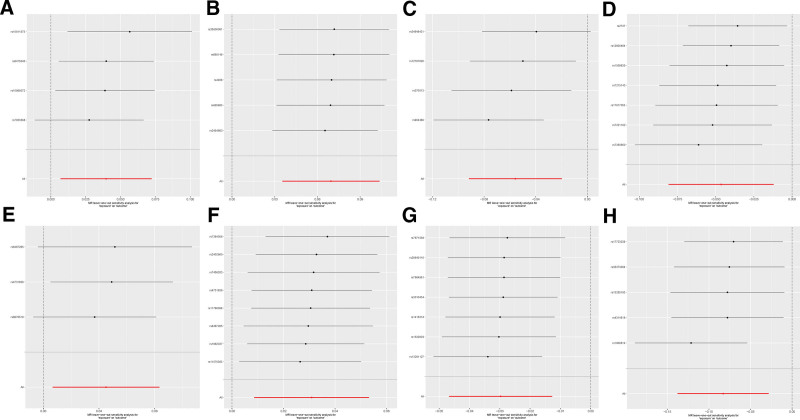
The forest plot of Leave-one-out analysis for the causal association between miRNAs and RA. (A) hsa-miR-31-5p, (B) hsa-miR-130a-3p, (C) hsa-miR-139-5p, (D) hsa-miR-152, (E) hsa-miR-182-5p, (F) hsa-miR-183-3p, (G) hsa-miR-204-5p, (H) hsa-miR-598. miRNA = microRNAs, RA = rheumatoid arthritis.

To ensure the robustness and stability of our findings, we conducted a comprehensive sensitivity analysis, including Cochran Q test, MR-Egger intercept test, and MR-PRESSO global test. All *P*-values of the above statistical tests were >.05 (with the exception of hsa-miR-182-5p, for which the MR-PRESSO global test was precluded due to methodological limitations arising from the small number of IVs), demonstrating the absence of significant heterogeneity or horizontal pleiotropy (Tables S2–4, Supplemental Digital Content, https://links.lww.com/MD/Q532). Additionally, we conducted the leave-one-out analysis to assess whether any SNPs exerted excessive influence on the MR results, and the analysis revealed that no individual SNP dominated the overall findings (Fig. 4). This comprehensive sensitivity analysis consistently supports the robustness and reliability of the study results.

### 3.2. Exploring the biological mechanisms and drug interactions of causal risk and protective miRNAs associated with RA

To explore the potential biological mechanisms underlying the causal miRNAs, we first conducted a literature review and found that among the 8 miRNAs identified, only hsa-miR-130a-3p^[35]^ and hsa-miR-204-5p^[10,36]^ had been experimentally validated in RA through miRNA microarray and/or RT-qPCR assays. Interestingly, the reported expression trends of these 2 miRNAs in circulating samples were consistent with our MR-based causal inferences, providing additional biological plausibility for their roles in RA pathogenesis. Consequently, we prioritized hsa-miR-130a-3p and hsa-miR-204-5p for subsequent bioinformatics analyses, including target gene prediction, ceRNA network construction, PPI network analysis, GO and KEGG functional enrichment, and druggable analysis.

#### 3.2.1. *Biological mechanisms and drug interactions of the RA risk factor has-miR-130a-3p*:

**Figure 5.**
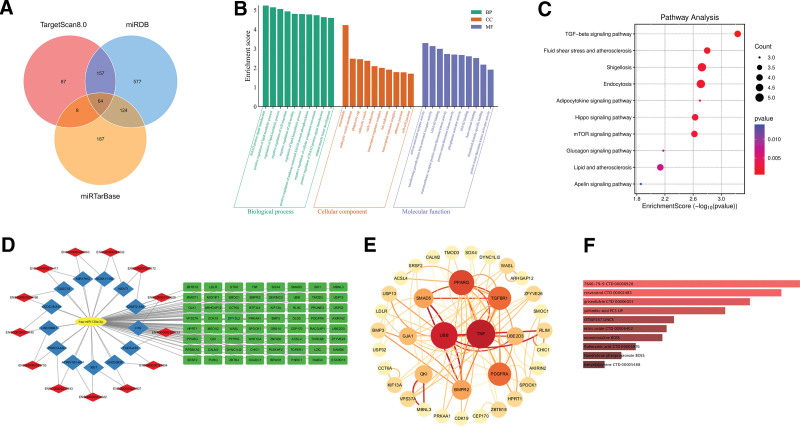
The potential biological mechanisms and drug interactions of hsa-miR-130a-3p. (A) The target genes of hsa-miR-130a-3p predicted by TargetScanHuman 8.0, miRDB, and miRTarBase databases. (B) GO analysis highlighted the significant biological processes, cellular components, and molecular functions that hsa-miR-130a-3p may modulate. (C) KEGG analysis revealed the key signaling pathways that hsa-miR-130a-3p potentially regulates. (D) Visualization of the lncRNA-miRNA-mRNA regulatory network: blue quadrilaterals represent lncRNAs, red diamonds indicate their corresponding Ensembl IDs, yellow ellipses denote miRNAs, and green rectangles represent mRNAs. (E) PPI network analysis revealed the core target genes regulated by hsa-miR-130a-3p. (F) Identification of the top ten drugs/compounds of the druggable genes ranked by *P*-values through the DSigDB tool in Enrichr. GO = gene ontology, KEGG = kyoto encyclopedia of genes and genomes, miRNA = microRNAs, PPI = protein–protein interaction.

A total of 64 putative target genes, 13 upstream lncRNAs, and 613 circRNAs were predicted to interact with hsa-miR-130a-3p (Fig. 5A, Table S5, Supplemental Digital Content, https://links.lww.com/MD/Q532). Given the excessive number and poor interpretability of predicted circRNAs, the circRNA-miRNA-mRNA network was not constructed. Instead, we focused on the lncRNA-miRNA-mRNA regulatory network (Fig. 5D). PPI network analysis based on STRING and visualized via Cytoscape identified TNF, UBB, PPARG, TGFBR1, and PDGFRA as central hub genes (Fig. 5E), suggesting their critical involvement in RA-related pathways.

GO enrichment analysis revealed that target genes of hsa-miR-130a-3p were primarily enriched in biological processes such as SMAD protein signal transduction, lipid metabolism regulation, and immune cell motility modulation (Fig. 5B). Cellular component enrichment highlighted microtubule structures and phagocytic vesicles, while molecular function analysis emphasized TGF-β receptor activity and protein serine/threonine kinase activity. KEGG pathway analysis further indicated significant enrichment in the TGF-β signaling pathway, Hippo signaling, and mTOR pathways, as well as pathways related to fluid shear stress, atherosclerosis, and adipocytokine signaling (Fig. 5C).

To identify potential therapeutic interventions, we cross-referenced predicted target genes with the DGIdb database and identified 20 druggable genes, including TNF, UBB, PPARG, and LDLR (Table S6, Supplemental Digital Content, https://links.lww.com/MD/Q532). Subsequent DSigDB-based drug screening via Enrichr identified resveratrol, flufenamic acid, and other candidate compounds (Fig. 5F). Notably, resveratrol has been previously reported as a natural agent with anti-inflammatory effects in RA models,^[37,38]^ while flufenamic acid is an NSAID that warrants further investigation for potential repurposing.^[39]^

#### 3.2.2. Biological mechanisms and drug interactions of the RA protective factor hsa-miR-204-5p:

**Figure 6.**
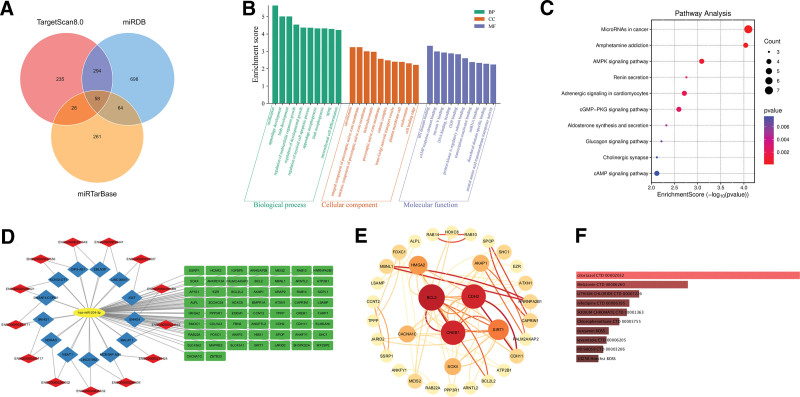
The potential biological mechanisms and drug interactions of hsa-miR-204-5p. (A) The target genes of hsa-miR-204-5p predicted by TargetScanHuman 8.0, miRDB, and miRTarBase databases. (B) GO analysis highlighted the significant biological processes, cellular components, and molecular functions that hsa-miR-204-5p may modulate. (C) KEGG analysis revealed the key signaling pathways that hsa-miR-204-5p potentially regulates. (D) Visualization of the lncRNA-miRNA-mRNA regulatory network: blue quadrilaterals represent lncRNAs, red diamonds indicate their corresponding Ensembl IDs, yellow ellipses denote miRNAs, and green rectangles represent mRNAs. (E) PPI network analysis revealed the core target genes regulated by hsa-miR-204-5p. (F) Identification of the top ten drugs/compounds of the druggable genes ranked by *P*-values through the DSigDB tool in Enrichr. GO = gene ontology, KEGG = kyoto encyclopedia of genes and genomes, miRNA = microRNAs, PPI = protein–protein interaction.

Target prediction identified 58 genes, 13 lncRNAs, and 377 circRNAs as potential downstream targets or upstream regulators of hsa-miR-204-5p (Fig. 6A, Table S7, Supplemental Digital Content, https://links.lww.com/MD/Q532). The constructed lncRNA-miRNA-mRNA network (Fig. 6D) and the PPI network highlighted BCL2, CREB1, CDH2, SIRT1, and HMGA2 as key hub genes (Fig. 6E).

GO analysis suggested that hsa-miR-204-5p may regulate ossification, mesenchymal differentiation, myeloid cell apoptosis, and organismal growth processes (Fig. 6B), potentially contributing to bone homeostasis and immune cell apoptosis in RA. Cellular component analysis indicated involvement in membrane rafts, chromatin structure, and vesicular transport, while molecular function enrichment highlighted transcriptional regulation and apoptosis-related pathways. KEGG pathway analysis revealed significant enrichment in AMPK, cAMP, and cGMP-PKG signaling pathways, as well as endocrine and metabolic regulatory pathways, including aldosterone synthesis and renin secretion (Fig. 6C).

Drug-target analysis identified 20 druggable genes (e.g., BCL2, CREB1, SIRT1) (Table S8, Supplemental Digital Content, https://links.lww.com/MD/Q532), and the DSigDB tool suggested that cilostazol, melatonin, and curcumin were among the top-ranked drug candidates (Fig. 6F). Literature evidence supports the anti-inflammatory and bone-protective effects of cilostazol,^[40–42]^ melatonin,^[43]^ and curcumin,^[44–46]^ highlighting their potential as adjunct therapies in RA management.^[38]^

## 4. Discussion

This study represents the first attempt to systematically investigate the causal effects of circulating miRNAs on RA risk using a 2-sample MR approach based on integrated GWAS and cis-miR-eQTL datasets.^[16]^ Our analysis identified 8 miRNAs with significant evidence supporting their potential causal roles in RA pathogenesis, including 4 risk-enhancing miRNAs (hsa-miR-31-5p, hsa-miR-130a-3p, hsa-miR-182-5p, and hsa-miR-183-3p) and 4 protective miRNAs (hsa-miR-139-5p, hsa-miR-152, hsa-miR-204-5p, and hsa-miR-598). Among these, hsa-miR-130a-3p^[35]^ and hsa-miR-204-5p^[10,36]^ demonstrated the strongest evidence, supported by both MR analysis and prior experimental validation in circulating samples from RA patients. Considering that the cis-miR-eQTLs data employed in our MR analysis were derived from blood-based studies, we propose that only miRNAs with both MR-based causal inference and circulating-level experimental validation should be considered high-confidence risk or protective factors for RA. Although hsa-miR-31-5p,^[9,47,48]^ hsa-miR-139-5p,^[49]^ hsa-miR-152,^[50,51]^ and hsa-miR-182-5p^[52]^ have shown differential expression in RA fibroblast-like synoviocytes or mesenchymal stem cells, their lack of validation at the circulating-level limits their current classification as high-confidence biomarkers. Notably, hsa-miR-183-3p and hsa-miR-598 remain experimentally uncharacterized in RA, suggesting their potential value for future functional studies.

To further elucidate the biological relevance of our findings, we focused subsequent bioinformatics analyses on hsa-miR-130a-3p and hsa-miR-204-5p. Our analyses revealed that hsa-miR-130a-3p potentially regulates 64 target genes enriched in the TGF-β, Hippo, and mTOR signaling pathways. Additionally, hsa-miR-130a-3p was found to interact with ceRNAs such as H19 and modulate key inflammatory and immune-related proteins including TNF, UBB, PPARG, and TGFBR1. Pharmacological assessment identified 20 druggable target genes and suggested therapeutic potential for agents such as resveratrol and flufenamic acid. Previous studies reported that hsa-miR-130a-3p is significantly elevated in the serum of RA patients, both pre-disease onset and in anti-citrullinated protein antibody-positive at-risk individuals, supporting its diagnostic and predictive biomarker potential.^[35]^ Moreover, mechanistic studies have shown that mechanical stress-induced downregulation of H19 in densely populated RA-FLSs leads to reduced miR-130a-3p levels and subsequent CDH11 upregulation, promoting synovial fibroblast invasiveness.^[53]^ Although the TGF-β,^[54–56]^ Hippo,^[57,58]^ and mTOR^[59,60]^ pathways have been extensively studied in RA, direct regulatory links between hsa-miR-130a-3p and these pathways remain to be clarified. Notably, resveratrol has been reported to modulate the TGF-β pathway in RA models,^[37]^ raising the intriguing possibility that its therapeutic effects may involve miR-130a-3p-mediated mechanisms. Further experimental studies are warranted to explore this hypothesis.

Similarly, hsa-miR-204-5p was identified as a putative regulator of 58 target genes enriched in AMPK, cGMP-PKG, and cAMP signaling pathways. This miRNA interacts with ceRNAs such as NEAT1 and NORAD and modulates critical RA-associated proteins including BCL2, SIRT1, and HMGA2. From a pharmacological perspective, hsa-miR-204-5p targets 20 druggable genes, with cilostazol, melatonin, and curcumin emerging as candidate therapeutic agents. Prior studies have reported that NORAD negatively regulates hsa-miR-204-5p, with elevated NORAD expression correlating with increased inflammatory markers such as TNF-α, IL-6, CRP, and ESR in RA patients.^[36]^ However, the downstream target genes of the NORAD/miR-204-5p axis remain largely undefined. Furthermore, NEAT1 has been shown to modulate inflammation in RA-FLSs by targeting hsa-miR-204-5p via the NF-κB signaling pathway,^[61]^ though the precise downstream mechanisms require further elucidation. The target prediction and enrichment results from our study provide a valuable reference for future functional validation of the ceRNA network centered on hsa-miR-204-5p and its role in RA pathogenesis. Notably, cilostazol, melatonin, and curcumin have demonstrated anti-inflammatory and immunomodulatory effects in RA models,^[42,62,63]^ suggesting potential avenues for miRNA-targeted therapeutic strategies.

While miRNAs have been extensively studied in the context of RA, most research has focused on synovial tissues and fibroblast-like synoviocytes, with relatively limited attention paid to circulating miRNAs.^[64]^ Current knowledge regarding the systemic regulatory roles, secretion dynamics, and diagnostic performance of circulating miRNAs in RA remains insufficient. Although several studies have reported altered circulating miRNA profiles in RA patients, the diagnostic sensitivity and specificity of these miRNAs remain suboptimal, with limited validation in large, multicenter cohorts. Moreover, the molecular mechanisms by which specific miRNAs modulate gene expression in peripheral blood mononuclear cells of RA patients are poorly understood.^[65]^ Emerging evidence also suggests that extracellular vesicles, including exosomes, play a crucial role in miRNA-mediated intercellular communication in RA. However, the mechanisms governing miRNA packaging, secretion, and functional transfer to target cells remain largely unexplored.^[66,67]^ Addressing these knowledge gaps will be essential for translating circulating miRNA biomarkers into clinical practice. In addition, investigating how existing anti-RA drugs modulate miRNA expression profiles may offer novel insights into drug repurposing and facilitate the development of miRNA-based therapeutic interventions. Furthermore, the advancement of targeted delivery systems for circulating miRNAs, such as lipid nanoparticles^[68]^ (LNPs) and engineered exosomes,^[67]^ presents a promising frontier for RA therapy.^[69]^ These strategies could enable precise modulation of disease-relevant miRNAs, thereby enhancing therapeutic efficacy while minimizing off-target effects.

In conclusion, our study provides novel evidence supporting the causal roles of specific circulating miRNAs in RA pathogenesis and highlights potential molecular targets and pharmacological agents for future therapeutic development. These findings lay a theoretical foundation for further mechanistic investigations and experimental validations, which are necessary to fully realize the clinical utility of miRNA-based diagnostics and therapeutics in RA.

## 5. Limitations

Despite offering valuable insights into the causal roles of circulating miRNAs in RA and elucidating their potential regulatory mechanisms, this study has several limitations that should be acknowledged. First, the genetic data used for both the exposure (cis-miRNA expression quantitative trait loci) and outcome (RA GWAS summary statistics) were derived exclusively from individuals of European ancestry. As a result, the generalizability of our findings to populations with different genetic backgrounds remains uncertain. Future studies should incorporate multi-ethnic cohorts to validate the observed associations. Second, due to the lack of individual-level data, we were unable to perform stratified analyses based on important demographic and clinical variables such as sex, ethnicity, age, and socioeconomic status. This limitation may obscure potential effect modifiers and population-specific risk profiles. Third, current cis-miR-eQTL resources are primarily derived from blood-based samples, with limited data available for other disease-relevant tissues such as synovial tissue. The absence of synovial cis-miR-eQTL data prevented us from assessing tissue-specific miRNA regulatory effects, which may play a critical role in RA pathogenesis. Fourth, while our bioinformatics analyses identified multiple target genes, signaling pathways, and druggable genes potentially regulated by hsa-miR-130a-3p and hsa-miR-204-5p, these mechanistic findings remain theoretical. Experimental validation, including in vitro and in vivo functional assays, is necessary to confirm the biological relevance and therapeutic potential of these miRNAs in RA. Finally, although 2 of the 8 miRNAs (hsa-miR-130a-3p and hsa-miR-204-5p) have been previously validated in clinical RA cohorts at the circulating level, the remaining miRNAs identified in this study lack experimental confirmation. Future prospective studies and well-designed functional experiments are warranted to address these gaps.

## 6. Conclusion

In summary, this study systematically investigated the causal relationships between circulating miRNAs and RA risk using a 2-sample MR framework. We identified 8 miRNAs with significant causal evidence, including 4 risk-enhancing and 4 protective miRNAs. Notably, the roles of hsa-miR-130a-3p as a risk factor and hsa-miR-204-5p as a protective factor were not only supported by MR analysis but also corroborated by previous clinical validation studies, thereby strengthening the credibility of our findings. Furthermore, through comprehensive bioinformatics analyses, we explored the potential molecular mechanisms, regulatory networks, and druggable targets associated with these key miRNAs. These findings provide novel theoretical insights into the pathogenesis of RA and highlight promising directions for the development of miRNA-based diagnostic tools and therapeutic strategies. Our study lays the groundwork for future experimental and clinical research aimed at translating these findings into clinical applications, ultimately contributing to precision medicine and improved patient outcomes in RA management.

## Acknowledgments

The authors thank FinnGen and Huan et al ([16] https://doi.org/10.1038/ncomms7601) for data sharing. Their contributions significantly facilitated the progress of this study.

## Author contributions

**Conceptualization:** Zehong Wei, Junping Yang.

**Data curation:** Zehong Wei, DongXu Chen, LianFa Li.

**Funding acquisition:** Zehong Wei, DongXu Chen, Junping Yang, Ying Wang.

**Investigation:** Zehong Wei, Ying Wang.

**Methodology:** Zehong Wei.

**Project administration:** DongXu Chen, Junping Yang.

**Software:** Zehong Wei, LianFa Li.

**Supervision:** LianFa Li, Junping Yang, Ying Wang.

**Validation:** DongXu Chen, LianFa Li, Junping Yang, Ying Wang.

**Visualization:** Zehong Wei.

**Writing – original draft:** Zehong Wei.

**Writing – review & editing:** Zehong Wei, Junping Yang, Ying Wang.

## Supplementary Material



## References

[1] Smolen JS Aletaha D Barton A. Rheumatoid arthritis. Nat Rev Dis Primers. 2018;4:18001. 29417936 10.1038/nrdp.2018.1

[2] Aletaha D Smolen JS. Diagnosis and management of rheumatoid arthritis: a review. JAMA. 2018;320:1360–72. 30285183 10.1001/jama.2018.13103

[3] Boeters DM Gaujoux-Viala C Constantin A Van Der Helm-Van Mil AHM. The 2010 ACR/EULAR criteria are not sufficiently accurate in the early identification of autoantibody-negative rheumatoid arthritis: results from the Leiden-EAC and ESPOIR cohorts. Semin Arthritis Rheum. 2017;47:170–4. 28601249 10.1016/j.semarthrit.2017.04.009

[4] Aletaha D Neogi T Silman AJ. 2010 rheumatoid arthritis classification criteria: an American College of Rheumatology/European League against rheumatism collaborative initiative. Ann Rheum Dis. 2010;69:1580–8. 20699241 10.1136/ard.2010.138461

[5] Sharma AR Sharma G Lee SS Chakraborty C. miRNA-Regulated Key components of cytokine signaling pathways and inflammation in rheumatoid arthritis. Med Res Rev. 2016;36:425–39. 26786912 10.1002/med.21384

[6] Chang C Xu L Zhang R. MicroRNA-mediated epigenetic regulation of rheumatoid arthritis susceptibility and pathogenesis. Front Immunol. 2022;13:838884. 35401568 10.3389/fimmu.2022.838884 PMC8987113

[7] Kmiołek T Paradowska-Gorycka A. miRNAs as biomarkers and possible therapeutic strategies in rheumatoid arthritis. Cells. 2022;11:452. 35159262 10.3390/cells11030452 PMC8834522

[8] Romo-García MF Bastian Y Zapata-Zuñiga M. Identification of putative miRNA biomarkers in early rheumatoid arthritis by genome-wide microarray profiling: a pilot study. Gene. 2019;720:144081. 31473322 10.1016/j.gene.2019.144081

[9] Tseng CC Wu LY Tsai WC. Differential expression profiles of the transcriptome and miRNA interactome in synovial fibroblasts of rheumatoid arthritis revealed by next generation sequencing. Diagnostics (Basel). 2019;9:98. 31426562 10.3390/diagnostics9030098 PMC6787660

[10] Wu LF Zhang Q Mo XB. Identification of novel rheumatoid arthritis-associated MiRNA-204-5p from plasma exosomes. Exp Mol Med. 2022;54:334–45. 35354913 10.1038/s12276-022-00751-x PMC8980013

[11] Richmond RC Smith GD. Mendelian randomization: concepts and scope. Cold Spring Harb Perspect Med. 2022;12 :a040501. 34426474 10.1101/cshperspect.a040501 PMC8725623

[12] Davey Smith G Hemani G. Mendelian randomization: genetic anchors for causal inference in epidemiological studies. Hum Mol Genet. 2014;23:R89–98. 25064373 10.1093/hmg/ddu328 PMC4170722

[13] Davies NM Holmes MV Smith GD. Reading Mendelian randomisation studies: a guide, glossary, and checklist for clinicians. Bmj. 2018;362:k601. 30002074 10.1136/bmj.k601 PMC6041728

[14] Smith GD Ebrahim S. “Mendelian randomization”: can genetic epidemiology contribute to understanding environmental determinants of disease? Int J Epidemiol. 2003;32:1–22. 12689998 10.1093/ije/dyg070

[15] Zheng J Baird D Borges MC. Recent developments in mendelian randomization studies. Curr Epidemiol Rep. 2017;4:330–45. 29226067 10.1007/s40471-017-0128-6 PMC5711966

[16] Huan T Rong J Liu C. Genome-wid*dentification of microRNA expression quantitative trait loci. Nat Commun. 2015;6:6601. 25791433 10.1038/ncomms7601 PMC4369777

[17] Boef AGC Dekkers OM Le Cessie S. Mendelian randomization studies: a review of the approaches used and the quality of reporting. Int J Epidemiol. 2015;44:496–511. 25953784 10.1093/ije/dyv071

[18] Yu J Meng S Xuan T. Identification of hsa-miR-193a-5p-SURF4 axis related to the gut microbiota-metabolites- cytokines in lung cancer based on Mendelian randomization study and bioinformatics analysis. Discov Oncol. 2024;15:475. 39331265 10.1007/s12672-024-01359-5 PMC11436685

[19] Burgess S Thompson SG; CRP CHD Genetics Collaboration. Avoiding bias from weak instruments in Mendelian randomization studies. Int J Epidemiol. 2011;40:755–64. 21414999 10.1093/ije/dyr036

[20] Burgess S Small DS Thompson SG. A review of instrumental variable estimators for Mendelian randomization. Stat Methods Med Res. 2017;26:2333–55. 26282889 10.1177/0962280215597579 PMC5642006

[21] Burgess S Dudbridge F Thompson SG. Combining information on multiple instrumental variables in Mendelian randomization: comparison of allele score and summarized data methods. Stat Med. 2016;35:1880–906. 26661904 10.1002/sim.6835 PMC4832315

[22] Verbanck M Chen CY Neale B Do R. Detection of widespread horizontal pleiotropy in causal relationships inferred from Mendelian randomization between complex traits and diseases. Nat Genet. 2018;50:693–8. 29686387 10.1038/s41588-018-0099-7 PMC6083837

[23] Hartwig FP Smith GD Bowden J. Robust inference in summary data Mendelian randomization via the zero modal pleiotropy assumption. Int J Epidemiol. 2017;46:1985–98. 29040600 10.1093/ije/dyx102 PMC5837715

[24] Agarwal V Bell GW Nam JW Bartel DP. Predicting effective microRNA target sites in mammalian mRNAs. Elife. 2015;4:e05005. 26267216 10.7554/eLife.05005 PMC4532895

[25] Chen Y Wang X. miRDB: an online database for prediction of functional microRNA targets. Nucleic Acids Res. 2020;48:D127–31. 31504780 10.1093/nar/gkz757 PMC6943051

[26] Cui S Yu S Huang HY. miRTarBase 2025: updates to the collection of experimentally validated microRNA-target interactions. Nucleic Acids Res. 2025;53:D147–56. 39578692 10.1093/nar/gkae1072 PMC11701613

[27] Chang L Xia J. MicroRNA regulatory network analysis using miRNet 2.0. Methods Mol Biol. 2023;2594:185–204. 36264497 10.1007/978-1-0716-2815-7_14

[28] Li JH Liu S Zhou H Qu LH Yang JH. starBase v2.0: decoding miRNA-ceRNA, miRNA-ncRNA and protein-RNA interaction networks from large-scale CLIP-Seq data. Nucleic Acids Res. 2014;42:D92–7. 24297251 10.1093/nar/gkt1248 PMC3964941

[29] Szklarczyk D Kirsch R Koutrouli M. The STRING database in 2023: protein-protein association networks and functional enrichment analyses for any sequenced genome of interest. Nucleic Acids Res. 2023;51:D638–46. 36370105 10.1093/nar/gkac1000 PMC9825434

[30] Shannon P Markiel A Ozier O. Cytoscape: a software environment for integrated models of biomolecular interaction networks. Genome Res. 2003;13:2498–504. 14597658 10.1101/gr.1239303 PMC403769

[31] Yu G Wang LG Han Y He QY. clusterProfiler: an R package for comparing biological themes among gene clusters. Omics. 2012;16:284–7. 22455463 10.1089/omi.2011.0118 PMC3339379

[32] Tang D Chen M Huang X. SRplot: a free online platform for data visualization and graphing. PLoS One. 2023;18:e0294236. 37943830 10.1371/journal.pone.0294236 PMC10635526

[33] Cannon M Stevenson J Stahl K. DGIdb 5.0: rebuilding the drug-gene interaction database for precision medicine and drug discovery platforms. Nucleic Acids Res. 2024;52:D1227–35. 37953380 10.1093/nar/gkad1040 PMC10767982

[34] Xie Z Bailey A Kuleshov MV. Gene set knowledge discovery with enrichr. Curr Protoc. 2021;1:e90. 33780170 10.1002/cpz1.90 PMC8152575

[35] Cunningham CC Wade S Floudas A. Serum miRNA signature in rheumatoid arthritis and “At-Risk Individuals.”. Front Immunol. 2021;12:633201. 33746971 10.3389/fimmu.2021.633201 PMC7966707

[36] Zhao X Lin W Zhou W. Clinical significance of long non-coding RNA NORAD in rheumatoid arthritis. Adv Rheumatol. 2024;64:9. 38238863 10.1186/s42358-024-00349-z PMC13488719

[37] Liu N Fan X Shao Y. Resveratrol attenuates inflammation and fibrosis in rheumatoid arthritis-associated interstitial lung disease via the AKT/TMEM175 pathway. J Transl Med. 2024;22:457. 38745204 10.1186/s12967-024-05228-1 PMC11095009

[38] Begh MZA Zehravi M Reza F. Therapeutic potential of phytocompounds in rheumatoid arthritis: molecular insights and clinical applications. Pathol Res Pract. 2025;269:155945. 40174276 10.1016/j.prp.2025.155945

[39] Zhang S Tang H Wang Y. Antibacterial and antibiofilm effects of flufenamic acid against methicillin-resistant Staphylococcus aureus. Pharmacol Res. 2020;160:105067. 32650057 10.1016/j.phrs.2020.105067

[40] Wahba MGF Messiha BS El-Daly ME Abo-Saif AA. Vardenafil and cilostazol can improve vascular reactivity in rats with diabetes mellitus and rheumatoid arthritis co-morbidity. Life Sci. 2019;229:67–79. 31085245 10.1016/j.lfs.2019.05.024

[41] Lee YS Lee SY Park SY Lee SW Hong KW Kim CD. Cilostazol add-on therapy for celecoxib synergistically inhibits proinflammatory cytokines by activating IL-10 and SOCS3 in the synovial fibroblasts of patients with rheumatoid arthritis. Inflammopharmacology. 2019;27:1205–16. 31123968 10.1007/s10787-019-00605-5

[42] Park SY Lee SW Shin HK. Cilostazol enhances apoptosis of synovial cells from rheumatoid arthritis patients with inhibition of cytokine formation via Nrf2-linked heme oxygenase 1 induction. Arthritis Rheum. 2010;62:732–41. 20131233 10.1002/art.27291

[43] Bagherifard A Hosseinzadeh A Koosha F. Melatonin and bone-related diseases: an updated mechanistic overview of current evidence and future prospects. Osteoporos Int. 2023;34:1677–701. 37393580 10.1007/s00198-023-06836-1

[44] Zhang C Weng Y Wang H. A synergistic effect of triptolide and curcumin on rheumatoid arthritis by improving cell proliferation and inducing cell apoptosis via inhibition of the IL-17/NF-κB signaling pathway. Int Immunopharmacol. 2024;142(Pt A):112953. 39226828 10.1016/j.intimp.2024.112953

[45] Pourhabibi-Zarandi F Rafraf M Zayeni H Asghari-Jafarabadi M Ebrahimi AA. The efficacy of curcumin supplementation on serum total antioxidant capacity, malondialdehyde, and disease activity in women with rheumatoid arthritis: a randomized, double-blind, placebo-controlled clinical trial. Phytother Res. 2024;38:3552–63. 38699839 10.1002/ptr.8225

[46] Gharat S Basudkar V Momin M. In-vitro and in-vivo evaluation of the developed curcumin-cyclosporine-loaded nanoemulgel for the management of rheumatoid arthritis. Immunol Invest. 2024;53:490–522. 38197806 10.1080/08820139.2024.2301997

[47] Wen JT Liu J Wan L. Triptolide inhibits cell growth and inflammatory response of fibroblast-like synoviocytes by modulating hsa-circ-0003353/microRNA-31-5p/CDK1 axis in rheumatoid arthritis. Int Immunopharmacol. 2022;106:108616. 35203042 10.1016/j.intimp.2022.108616

[48] Xin P Tan Z Wang Z Chen Y Zhuang Y. Circular RNA hsa_circ_0000175 serves as a potential biomarker for rheumatoid arthritis via miR-31-5p/GSDME Axis. Biochem Genet. 2024;62:2522–39. 37968534 10.1007/s10528-023-10576-6

[49] Hyland M Mennan C Davies R. Extracellular vesicles derived from umbilical cord mesenchymal stromal cells show enhanced anti-inflammatory properties via upregulation of miRNAs after pro-inflammatory priming. Stem Cell Rev Rep. 2023;19:2391–406. 37474869 10.1007/s12015-023-10586-2 PMC10579155

[50] Jiang H Liu J Fan C Wang J Li W. lncRNAS56464.1 as a ceRNA promotes the proliferation of fibroblast‑like synoviocytes in experimental arthritis via the Wnt signaling pathway and sponges miR‑152‑3p. Int J Mol Med. 2021;47 :17. 33448322 10.3892/ijmm.2021.4850 PMC7834957

[51] Miao CG Qin D Du CL. DNMT1 activates the canonical Wnt signaling in rheumatoid arthritis model rats via a crucial functional crosstalk between miR-152 and the DNMT1, MeCP2. Int Immunopharmacol. 2015;28:344–53. 26093272 10.1016/j.intimp.2015.06.013

[52] Ou-Yang Y Dai MM. Screening for genes, miRNAs and transcription factors of adipogenic differentiation and dedifferentiation of mesenchymal stem cells. J Orthop Surg Res. 2023;18:46. 36647068 10.1186/s13018-023-03514-0 PMC9843867

[53] Janczi T Böhm B Fehrl Y. Mechanical forces trigger invasive behavior in synovial fibroblasts through N-cadherin/ADAM15 -dependent modulation of LncRNA H19. Sci Rep. 2025;15:9814. 40118917 10.1038/s41598-025-94012-2 PMC11928650

[54] Wu G Su T Zhou P. Engineering M2 macrophage-derived exosomes modulate activated T cell cuproptosis to promote immune tolerance in rheumatoid arthritis. Biomaterials. 2025;315:122943. 39509857 10.1016/j.biomaterials.2024.122943

[55] Mao X Wu W Nan Y Sun W Wang Y. SMAD2 inhibits pyroptosis of fibroblast-like synoviocytes and secretion of inflammatory factors via the TGF-β pathway in rheumatoid arthritis. Arthritis Res Ther. 2023;25:144. 37559090 10.1186/s13075-023-03136-1 PMC10410963

[56] Cutolo M Campitiello R Gotelli E Soldano S. The role of M1/M2 macrophage polarization in rheumatoid arthritis synovitis. Front Immunol. 2022;13:867260. 35663975 10.3389/fimmu.2022.867260 PMC9161083

[57] Wang T Wang Z Qi W Jiang G Wang G. Possible future avenues for rheumatoid arthritis therapeutics: hippo pathway. J Inflamm Res. 2023;16:1283–96. 36998323 10.2147/JIR.S403925 PMC10045326

[58] Du Y Cui R Tian N Chen M Zhang XL Dai SM. Regulation of type I interferon signature by VGLL3 in the fibroblast-like synoviocytes of rheumatoid arthritis patients via targeting the Hippo pathway. Arthritis Res Ther. 2022;24:188. 35941675 10.1186/s13075-022-02880-0 PMC9358906

[59] Liu H Chen Y Huang Y. Macrophage-derived mir-100-5p orchestrates synovial proliferation and inflammation in rheumatoid arthritis through mTOR signaling. J Nanobiotechnology. 2024;22:197. 38644475 10.1186/s12951-024-02444-1 PMC11034106

[60] Zhang M Iwata S Sonomoto K. mTOR activation in CD8+ cells contributes to disease activity of rheumatoid arthritis and increases therapeutic response to TNF inhibitors. Rheumatology (Oxford). 2022;61:3010–22. 34791054 10.1093/rheumatology/keab834

[61] Xiao J Wang R Zhou W Cai X Ye Z. LncRNA NEAT1 regulates the proliferation and production of the inflammatory cytokines in rheumatoid arthritis fibroblast-like synoviocytes by targeting miR-204-5p. Hum Cell. 2021;34:372–82. 33394349 10.1007/s13577-020-00461-4

[62] Zhao CN Wang P Mao YM. Potential role of melatonin in autoimmune diseases. Cytokine Growth Factor Rev. 2019;48:1–10. 31345729 10.1016/j.cytogfr.2019.07.002

[63] Park C Moon DO Choi IW. Curcumin induces apoptosis and inhibits prostaglandin E(2) production in synovial fibroblasts of patients with rheumatoid arthritis. Int J Mol Med. 2007;20:365–72. 17671742

[64] Ma X Taxi W Guo Y. A bibliometric analysis of miRNAs in rheumatoid arthritis from 2001 to 2022: research hotspots and trends. Int J Rheum Dis. 2024;27:e15121. 38562078 10.1111/1756-185X.15121

[65] Ebrahimian H Akhtari M Akhlaghi M. Altered expression of apoptosis-related genes in rheumatoid arthritis peripheral blood mononuclear cell and related miRNA regulation. Immun Inflamm Dis. 2023;11:e914. 37506143 10.1002/iid3.914 PMC10336681

[66] Ahmed SF Jasim SA Pallathadka H. New therapeutic strategies for the inflammatory rheumatoid arthritis disease: emphasizing mesenchymal stem cells and associated exo-miRNA or exo-lncRNA. Cell Biochem Biophys. 2024;82:1599–611. 38822204 10.1007/s12013-024-01316-7

[67] Abebaw D Akelew Y Adugna A. Extracellular vesicles: immunomodulation, diagnosis, and promising therapeutic roles for rheumatoid arthritis. Front Immunol. 2024;15:1499929. 39624102 10.3389/fimmu.2024.1499929 PMC11609219

[68] Sharma S Ghosh R Marianesan AB Hussain S Pandey JD Kumar M. Nanostructured lipid carriers in rheumatoid arthritis: treatment, advancements and applications. Inflammopharmacology. 2025;33:941–58. 40025299 10.1007/s10787-025-01669-2

[69] Liu Y Jiang P Qu Y. Exosomes and exosomal miRNAs: a new avenue for the future treatment of rheumatoid arthritis. Heliyon. 2024;10:e28127. 38533025 10.1016/j.heliyon.2024.e28127 PMC10963384

